# A Scoping Review of Choice Architecture to Promote Healthy Nutrition in Health and Care Settings

**DOI:** 10.1111/jhn.70111

**Published:** 2025-08-25

**Authors:** Victoria Bion, Grace Turner

**Affiliations:** ^1^ London School of Hygiene and Tropical Medicine London UK; ^2^ NIHR Health Protection Research Unit in Environmental Change and Health, London School of Hygiene and Tropical Medicine London UK

**Keywords:** behaviour change, choice architecture, diet, health and care settings, plant‐based diets

## Abstract

**Introduction:**

Poor diets are a remediable risk factor for non‐communicable diseases. Sickness absence rates for national health service (NHS) staff are substantially higher than the public sector average (5.6% vs. 3.6%). Hospital inpatients are often being treated for the downstream consequences of poor diets. Systematic reviews and meta‐analyses support national health recommendations for plant‐based diets that emphasise consumption of varied whole plant‐source foods with minimal consumption of animal products. These diets are increasingly recognised as compatible with planetary health and are associated with lower greenhouse gas emissions. There is increasing interest in using choice architecture interventions (subtly changing the environment in which individuals make decisions) to encourage healthier plant‐based food choices in health and care settings. This approach may prove cost effective, encouraging better choices for staff and inpatients with minimal upfront investment.

**Objective:**

To summarise evidence for choice architecture interventions aimed at changing dietary choices made by staff and inpatients in high‐income health and care settings. This review aims to inform decision making on food service provision in health and care organisations.

**Methods:**

Medline, CINAHL PLUS, GreenFile, and Web of Science were searched for studies which examined choice architecture on dietary choices in high‐income health and care settings. Studies referenced in systematic reviews were examined for inclusion from 4th to 10th June 2024. A typology used in a previous review conducted by Public Health England (categories: availability, positioning, pricing, functionality, presentation, information, sizing) was modified to include a category for using defaults (the action that occurs when no choice is made). Randomised experimental, quasi‐experimental, interrupted time series and before and after studies reporting on nutritional measures or a measure of healthy food purchases were included. Studies from non‐healthcare settings were excluded.

**Results:**

None of the studies explicitly encouraged plant‐based diets or measured environmental impact although 12 studies measured change in plant‐based choices using measures such as fruit and vegetable servings. A total of 51 studies were included focused on other healthy dietary interventions. A total of 31 of these studies implemented more than one type of choice architecture. Twenty studies were conducted in cafeterias, eight on hospital vending machines, six in hospital retail stores, three in residential care homes, and five in an inpatient setting. A further nine studies either implemented changes across multiple aspects of healthcare food provision, included non‐healthcare workplaces, or examined hospital office‐based interventions. Overall, 34 of the 51 studies reported a positive change in healthy food choices and only six studies reported no significant change or an adverse change. Availability, pricing and positioning of items are associated with a change in dietary choices. Evidence for informational changes was mixed and at worst, had a negative reaction, increasing unhealthy purchasing. Few studies used sizing or presentation elements and none of the studies evaluated functionality or default elements. None of the inpatient studies examined persistent change in dietary choices for long‐term health.

**Conclusions:**

In this review, the evidence indicates that choice architecture interventions can support healthier food choices in health and care settings. However, there is limited research and nutritional evaluation of choice architecture interventions that encourage plant‐based diets. Further well‐conducted studies are needed in health and care settings to determine optimal typologies, or combined approaches, for making healthier dietary choices. Given the established evidence of plant‐based diets for long‐term health, and the lower environmental impact of these diets, studies using choice architecture to encourage plant‐based choices in health and care settings should be conducted and should evaluate nutritional, financial, and environmental outcomes. The effectiveness of choice architecture techniques in inpatient catering to encourage and role model healthier diets should be investigated to tackle dietary inequality and the burden of diet‐related chronic disease.

## Summary

- Choice architecture can improve healthy food choices cost‐effectively, especially through changes to availability, pricing, and positioning.
- There is a critical evidence gap on using choice architecture to promote plant‐based diets and assess environmental impact.
- Inpatient and care home settings are under‐researched but present unique opportunities to support behaviour change.

## 1 Introduction

### 1.1 Preventative Healthcare Systems

A total of 40% of UK adults have at least one long‐standing chronic condition [1]. Good nutrition is critical to preventing chronic disease: Diet‐related ill‐health contributes 10% of morbidity and mortality in the UK, a burden that disproportionately affects deprived groups [2]. Progress on reducing risk factors such as smoking and cholesterol have been offset by increasing obesity [3]. Social deprivation is associated with a higher risk of emergency hospital admission, poor diet, and non‐communicable diseases (NCDs): those living in deprived areas are almost four times more likely to die prematurely from cardiovascular disease compared to the least deprived [4, 5, 6].

Healthcare organisations play an important role in promoting good nutrition. They can role model healthy diets through their free or discounted food provision for staff, visitors and patients, and influence wider communities. The UK National Health Service (NHS) employs 1.5 million staff, the largest employer in Europe [7]. Providing 24‐h access to healthy food is essential given the elevated risk of cancer, cardiovascular disease and Type II diabetes mellitus (T2DM) among shift workers. Sickness absence rates in the NHS exceed the public sector average (5.6% vs. 3.6%) [8, 9]. Yet, as highlighted in an independent review of NHS food, 39% of staff felt that the food provision was poor, and night‐shift staff often lacked any healthy or hot options overnight [10].

Inpatient catering also offers an opportunity to optimise nutrition and diet. During major life events patients may be more receptive adopting healthy behaviours that are sustained after discharge [11]. Over 140 million meals are served, free of charge, to NHS patients annually [10]. Healthcare organisations can contribute to addressing health inequalities by improving access to healthy food in their patient population. However, hospital food is often perceived and portrayed as unpalatable [10]. A Patients' Association survey reveals that 50% of patients felt poor presentation of food negatively impacted their intake, some being served food that was still partially frozen, or reporting lack of access to, and choice of, healthy options, particularly fresh fruit [12]. Inadequate choice, and presentation of patient food, therefore, presents an opportunity for intervention.

### 1.2 Motivation and Environmental Stewardship

UK dietary surveys report excessive intake of fat, salt and sugar, and too little fibre, fruit and vegetables [13]. National dietary guidelines recommend following a predominantly whole, plant‐based diet (which does not need to exclude animal‐based foods completely) to lower risk of NCDs such as obesity, T2DM and cardiovascular disease [14, 15]. Plant‐based foods also have a lower carbon footprint than animal products [16]. Compared to current diets, the UK Eatwell guide has lower associated greenhouse gas emissions and land use [17]. Environmental concern may be an increasingly important motivator for behaviour change towards healthier and more sustainable plant‐based diets. A total of 25% of British adults are concerned about climate change [18]. In a European survey, nearly half of UK respondents reported reducing meat intake citing health (48%) and environmental (29%) concerns [19]. Finally, NHS organisations are committed to supporting Net Zero targets. NHS food is estimated to contribute 1540ktCO_2_e (kilotonnes of carbon dioxide equivalents) annually—6% of total emissions, representing an important lever to simultaneously meet health and environmental goals [20].

### 1.3 Choice Architecture: An Inclusive Approach?

The Centre for Climate Change and Social Transformations review concluded that to be effective, educational interventions should be combined with approaches to make “plant‐based foods more available, convenient, attractive and affordable” [21]. Governments have historically focused on high‐agency behaviour change interventions (e.g., awareness campaigns) that require personal resources to benefit [22]. Conversely, low‐agency interventions such as manufacturer‐implemented salt reductions (macroenvironment), or prominent supermarket placement of healthier options (microenvironment i.e., the immediate environment in which the choice occurs) are found to be more effective and equitable [23, 24].

Low‐agency interventions in microenvironments are often termed “nudge” or “choice architecture” interventions based on philosophical principles of libertarian paternalism—the idea that public of private institutions can influence behaviour and simultaneously preserve freedom of choice [25]. Choice architecture, defined here as designing the microenvironment to promote certain choices, can be an appealing concept to decision makers, achieving desired behaviour change while seemingly avoiding negative publicity associated with restricting choice [25]. This is particularly relevant for food choices. Food preferences are highly individual. With increasingly diverse populations, hospitals need to provide culturally appropriate meals when encouraging healthy foods [26].

A 2019 systematic review found evidence of effective dietary choice architecture interventions in healthcare staff, however, the diets promoted focussed predominantly on reducing calorie intake, rather than encouraging plant‐based choices [27]. Additionally, this evidence may not be generalisable to inpatients who often experience low appetite and may have specific nutritional needs. Studies in non‐healthcare settings such as workplaces and educational institutions suggest that choice architecture interventions are a promising way to increase healthier food choices [28, 29].

Conditional funding (CQUIN) in England incentivises healthcare providers to reduce availability and promotion of high fat, sugar and salt foods: although incentivising plant‐based foods has not been implemented [30]. British Dietetic Association (BDA) sustainability guidance recommends using choice architecture to increase consumption of plant‐based diets but it is unclear if this is being implemented [31]. As anchor institutions, healthcare organisations are well placed to improve the nutrition of patients, staff, and the communities they serve.

### 1.4 Aims

This scoping review aims to examine the evidence for choice architecture interventions to improve dietary behaviours of staff and patients in health and care settings. A secondary aim was to determine the evidence for use of choice architecture interventions specifically encouraging plant‐based diets.

## 2 Methods

A literature search was conducted from 4th to 10th June 2024 across Medline, CINAHL PLUS, GreenFile and Web of Science using search concepts and terms (Table 1; final search strategy in Appendix 10). Health promotion and health behaviour terms were included to provide a more comprehensive search, as prior literature suggests the term “choice architecture” is often inconsistently used, and poorly specified [32]. Reference lists of identified systematic reviews were scanned for any additional studies.

**Table 1:** Search concepts and terms used to develop the search strategy.

Search concept	Search terms
Choice Architecture	Health Promotion OR Health Behaviour OR Choice Behaviour OR Choice Intervention OR Food Preference OR Nudge OR Choice Architecture OR Decision Making
Diet	Diet OR Plant‐based OR Mediterranean OR vegetarian OR vegan OR dietary pattern OR healthy diet OR food OR meals
Health or Care	Patients OR Hospitals OR home care services OR residential facilities OR residential care OR nursing home OR healthcare

Studies met the inclusion criteria if they fulfilled all of the following:

### 2.1 Study Design

Experimental or quasi‐experimental studies, interrupted time series and before and after studies were included.

### 2.2 Participants

Hospital or care‐home staff, inpatients, or visitors in high‐income countries (as classified by the World Bank) were included. Increasing plant‐based diets is predominantly a concern in high‐income settings, where overconsumption of animal‐source foods and the associated environmental and health impacts are more relevant, and institutional food service systems differ substantially from those in lower‐income countries.

### 2.3 Interventions

Behavioural interventions were included based on the TIPPME tool (typology of interventions in proximal physical micro‐environments) [33]. TIPPME outlines six ways to alter properties or placement of objects, or stimuli in the proximal micro‐environment which can be applied to behaviours. Examples include decreasing availability or size of undesirable choices or labelling items (e.g., traffic‐light labelling). To remain consistent with a previous Public Health England (PHE) systematic review on choice architecture in NHS hospitals, pricing interventions were included if applied in the immediate setting in which the choice was being made [27]. For example, discounting of heathier option or “meal deals” at the canteen level was included, whereas economic policies applied more broadly e.g., sugar taxation were excluded. This approach was used to ensure the review remained consistent with a previous PHE systematic review on choice architecture in NHS hospitals [27, 34]. Additionally, a category for default options was included after discussion with one of the authors of the TIPPME tool, given the recent New York case study [35]. The most recent TIPPME typology published by Hollands et al. [33] was used resulting in minor differences from the PHE review.

### 2.4 Outcomes

Studies were included if they reported nutritional measures (such as changes to BMI, macronutrient composition, or food groups) or a measure of healthy food purchases as defined by study authors. Given the broad umbrella of dietary patterns encompassed by the term “plant‐based”, all stated definitions of “healthier” foods or meals were included and the definition extracted to determine which dietary choices were promoted by the intervention. Interventions that promoted plant‐source foods (such as fruit, vegetables, wholegrains, pulses, legumes) or high fibre content were considered in‐keeping with encouraging healthier diets. Secondary outcomes of interest were environmental measures such as CO_2_e, or metrics on land and water use.

Following database searching, titles and abstracts were retrieved and screened for relevance by a single review author (VB). Limits included English language, and from 2000 onwards to ensure relevance to the current health services. Studies from non‐high‐income countries, qualitative studies, and behavioural interventions implemented outside the immediate environment where the target behaviour was performed (e.g., lifestyle advice, education programmes) were excluded. Public service institutions such as schools or prisons were excluded. Following preliminary screening, full text records were obtained and assessed for inclusion or exclusion using the inclusion criteria above. Data extraction was standardised using a predefined excel template to include details on methods, participants, intervention and mapping to TIPPME domains, and outcomes, and where relevant, reason for exclusion was recorded.

Results were narratively synthesised due to substantial heterogeneity in design and intervention type. The PRISMA‐ScR checklist was used to guide reporting (Appendix 11).

## 3 Results

**Figure 1:**
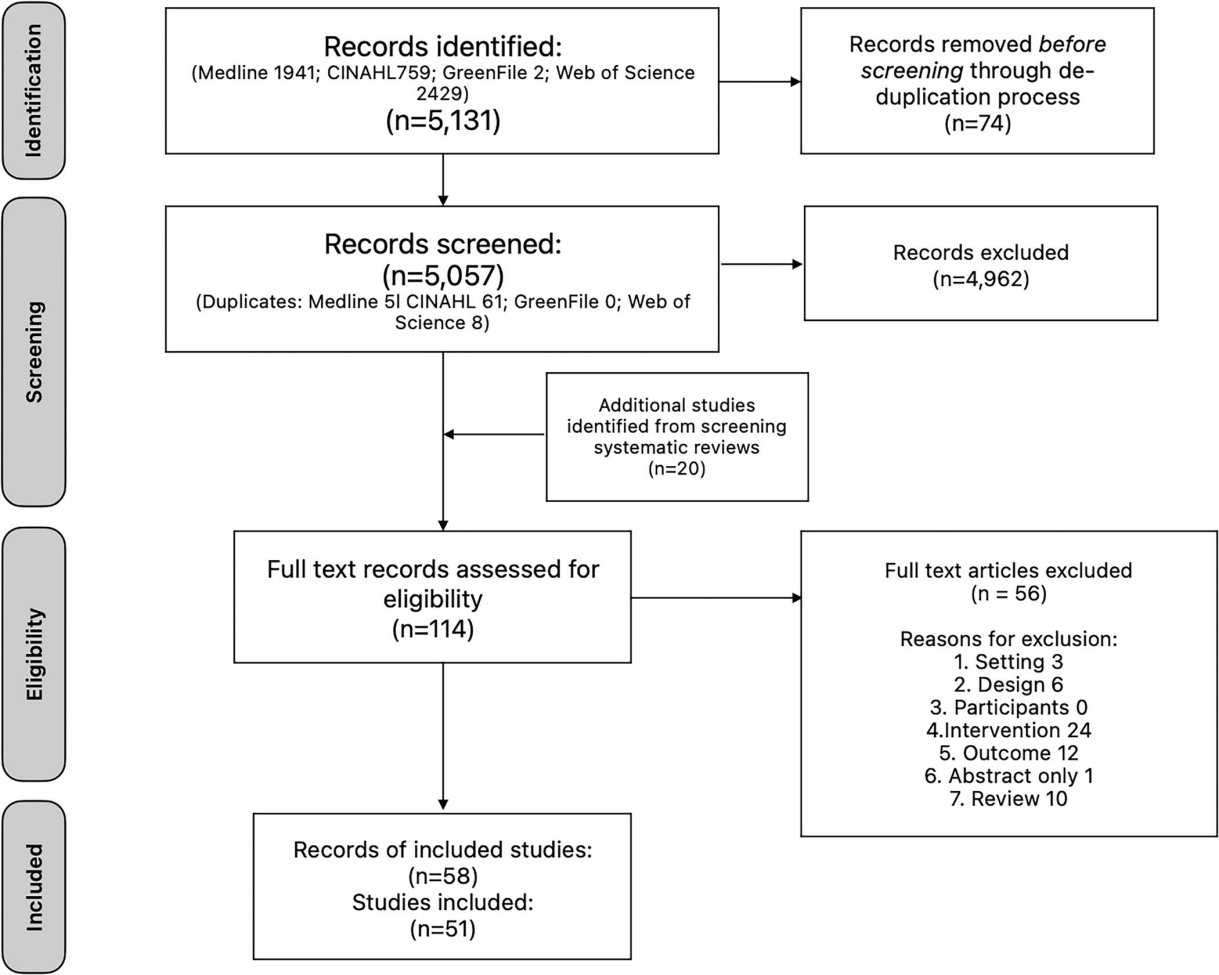
PRISMA‐ScR flow diagram for the scoping review process.

Of 5131 hits providing 5057 unique records, 58 met the criteria. Ten records described partial or interim results from three studies resulting in 51 studies for review (Figure 1).

### 3.1 Setting

Of the 51 studies, 19 were conducted in the US [36, 37, 38, 39, 40, 41, 42, 43, 44, 45, 46, 47, 48, 49, 50, 51, 52, 53, 54, 55, 56, 57, 58, 59, 60, 61], eight in the UK [62, 63, 64, 65, 66, 67, 68, 69], eight in the Netherlands [70, 71, 72, 73, 74, 75, 76, 77], and seven in Australia [78, 79, 80, 81, 82, 83, 84]. The remainder took place in Canada [85, 86, 87], Denmark [88, 89], Ireland [90], Japan [91], New Zealand [92] and Norway [93]. Details on study design and characteristics are provided in Appendix 3.

Most studies examined hospital cafeterias *n* = 24 [36, 37, 38, 39, 40, 41, 42, 43, 44, 45, 46, 47, 48, 49, 50, 51, 52, 53, 61, 62, 73, 74, 75, 76, 77, 78, 79, 86, 87, 88, 90], vending machines *n* = 8 [54, 63, 64, 65, 66, 67, 80, 92], or retail outlets *n* = 6 [59, 60, 68, 69, 84, 91]. One study [70] examined meeting rooms and four studies [55, 56, 82, 83] implemented changes across more than one setting. A total of 43 studies conducted staff interventions, 24 of which examined sites where visitors were included in the analysis but did not disaggregate data by specific user group limiting conclusions on differential effects of interventions on staff versus visitors. Three studies [57, 58, 93] focused on nursing home residents and five studies [71, 72, 81, 85, 89] targeted hospital inpatients. None of the studies examined plant‐based foods or measured environmental impact. Only one study used behaviour change frameworks to structure their intervention [91]. Choice Architecture elements used by studies are presented in Table 2.

**Table 2:** TIPPME classification of study interventions and reported direction of effect.

Study	Typology							Reported effect
	Availability	Functionality/Defaults	Information	Positioning	Presentation	Pricing	Sizing	
Hospital cafeteria								
Whitt [36]								
Ryan [78]								
Thorndike [37, 38, 41, 44, 45]								
Thorndike [40]								
Levy [39, 42, 43, 61]								
Block [46]								
Lowe [47]								
Warsaw [48]								
Sato [49]								
Mah [87]								
Meeusen [77]								
Mazza [50]								
Webb [51]								
Patsch [52]								
Vanderlee [86]								
van Kleef [76]								
Geaney [90]								
Dorresteijn [75]								
Lassen [88]								
MacDonald [79]								
Hospital vending								
Gorton [92]								
Grivois‐Shah [54]								
Campbell [63]								
Pechey [64]								
Boelsen‐Robinson [80]								
Griffiths 2024 [66]								
Griffiths 2020 [65]								
Public Health England [67]								
Hospital retail								
Racette [59]								
Kawabata [91]								
Allan [68]								
Elbel [60]								
Blake [84]								
Simpson [69]								
In‐patients								
Barrington [81]								
Holst [89]								
Doorduijn [72]								
Basak [85]								
van der Zanden [71]								
Nursing homes								
Crogan [57]								
Hansen [93]								
Remsburg [58]								
Others (meeting rooms, mixed setting, multiple worksites)								
Immink [70]								
Epel [55]								
LaCaille [56]								
Tinney [82]								
Walker [83]								
Kwak [74]								
Vermeer [73]								
Holdsworth [62]								
Beresford [53]								

*Note:* Classification from Hollands et al. [33] with the additional elements of pricing and defaults. None of the studies implemented interventions on functionality or defaults. Typologies employed by studies are highlighted in blue. Reported effect: **Green** = positive result reported for primary outcome; **Amber** = mixed effects: null result reported for primary outcome, some positive secondary outcome findings; **Red** = negative: null result or undesirable change reported.

### 3.2 Hospital Cafeteria

Twenty studies exclusively examined hospital cafeterias, including two in paediatric settings [36, 50]. Most studied staff, with some including visitors. Thirteen studies implemented multiple choice architecture elements. The most common elements were information (19 studies), followed by availability, position, and pricing. Sixteen studies measured purchasing, two measured dietary consumption, one study looked at both, and one measured purchasing and biomarkers such as BMI.

#### 3.2.1 Single Element Interventions

Two studies implemented only item labelling [36, 49]. A paediatric hospital café implemented traffic‐light labelling to all items based on criteria such as having fruits, vegetables, wholegrains, lean protein or low‐fat dairy as the main component [36]. Negative criteria included high saturated fat or high calorie items. After a washout period a cartoon label was added to green items and flyers explained the labelling system. Traffic‐light labelling led to a 7% reduction in the proportion of unhealthy food purchases (*p* < 0.001), however, cartoon labelling increased unhealthy purchases by 5% (*p* = 0.057). Sato et al. [49] analysed and modified cafeteria entrees ensuring a “healthy pick” entrée was available daily (labelled to contain < 35% of calories from fat, 10% of calories from saturated fat, and < 1000 mg of sodium per entrée). A non‐significant positive trend was seen towards increasing healthy pick sales, but bias risk was high due to confounding increases in entrée price during the study.

Two Australian studies examined changing food availability [78, 79]. A retrospective before and after study classified beverages as red, yellow, or green using criteria such as calorie and fruit content [78]. Customers were unaware of the classifications. All red beverages were removed from display and made available on request. Purchasing data revealed a 23% decrease in red beverages sold (*p* < 0.001), an 18% increase in amber (*p* = 0.001), and a 5% increase in green (*p* = 0.438). Eighteen‐month follow‐up data confirmed sustained change: 7% red (*p* = 0.002), 55% amber (*p* = 0.026) and 38% green (*p* = 0.025). Surveys revealed support for the initiative but stressed personal choice. Limitations include lack of control, few data points (18‐month gap), no trends analysis, no assessment of compensatory behaviour elsewhere, and a low 49% survey response rate. MacDonald et al. [79] introduced health catering toolkits to local caterers at aboriginal community‐run sites including traffic‐light procurement guides requiring at least 50% of procurement to come from the “best choice” category while limiting high energy, saturated fat, sugar and salt foods. Catering receipts demonstrated “foods to limit” decreased from 51% to 1% at one site, and 32% to 6% at another, while “best choice” foods from 18% to 24% and 17% to 33%, respectively. The study was high risk for bias as other changes made at the sites were not considered, catering receipts were only available from two sites out of the five, and measurement relied on subjective portion size estimates.

One study solely examined pricing. Warsaw et al. [48] found that discounting healthier items (e.g., salad or bottled water) increased healthy purchases and decreased unhealthy purchases. However, taxing unhealthier items had mixed effects with cheeseburger purchases increasing.

#### 3.2.2 Multi‐Element Interventions

There were six labelling‐predominant studies [39, 42, 43, 47, 50, 51, 61, 86, 88]. Thorndike et al. [39, 42, 43, 61] conducted an interrupted time series study using traffic‐light labelling and positioning to promote healthier choices. Purchasing data demonstrated a decrease in calories per transaction at 1‐ and 2‐year post‐intervention (−19 and −35 kcal, respectively). Sub‐group analysis demonstrated similar effects across ethnicities and job types. A similar RCT by Lowe et al. [47] increased availability of less energy dense foods and applied traffic‐light labelling. Authors reported similar decreases in purchased calories although selective outcome reporting, a high attrition rate, and lack of control increases risk of bias. An interrupted time series analysis of a phased intervention over 21 months implemented traffic‐light labelling and pricing, sequentially adding additional interventions (use of emoticon‐labelling, colour label grouping, and health social norm messaging) [50]. Compared to price changes alone, traffic‐light labelling was associated with a 2.9% increase in healthy beverage sales (*p* < 0.0001). Further interventions had a negative impact, in some cases reversing the positive traffic‐light effects.

Two studies examined calorie labelling, demonstrating a shift towards lower‐calorie options and reduced self‐reported sodium, saturated fat, and total fat intake compared with control after adjustment for confounding factors [51, 86]. Denmark researchers trialled a widely recognised “healthy symbol” [88]. Nutritional intake was estimated from plate photographs showing significantly lower energy, fat and refined sugar intake at 6 weeks and 6 months compared to control. They also noted a significantly greater increase in fruit and vegetable consumption in the intervention group. Whilst the control canteen differed in serving system and pricing, this study used a more accurate dietary intake measure than purchasing data alone.

Five North American studies primarily explored pricing [37, 38, 40, 41, 44, 45, 46, 52, 87]. In an extension of previous work which introduced food labelling and positioning changes, Thorndike et al. [40] conducted an RCT, randomising groups to peer comparison feedback letters, financial incentives plus feedback, or no intervention Purchasing data from 2627 employees demonstrated increased healthy “green” purchases during the 3‐month intervention for the incentives‐plus‐feedback group compared to control (2.2% vs. 0.1%, *p* = 0.03). A later RCT added twice weekly personalised emails to the social norm letters and financial incentives for 12 months [37, 38, 41, 44, 45]. Follow up of 602 patients for a further 12‐month post‐intervention included weight measurement as well as purchasing data. Purchasing data corroborated the previous trial with a larger percentage change. Compared with baseline, the intervention group increased healthy purchases by 7.3% (95% CI: 5.4% to 9.3%), reduced unhealthy purchases by 3.9% (95% CI: −5.0 to −2.7%), and decreased calories purchased by 49.5 kcal/day (95% CI: −75.2 to −23.9) more than control. However, weight change was not significant at 12‐ or 24‐month assessment.

Block et al. [46] conducted a phased non‐randomised trial increasing sugar sweetened beverage (SSB) price and providing educational messaging. Comparison with control was inconsistently applied, however, when SSB price increased either alone or in combination with educational messaging, SSB sales decreased by 26% and 36%, respectively, from baseline. Two further studies introduced price changes alongside point‐of‐sale nutrition information and demonstrated associated increases in healthier purchasing [52, 87]. Although lacking a comparator to assess underlying trends and seasonal variation, Patsch et al. [52] recorded an 8% increase in gross monthly sales, and a four‐ to eightfold increase in profit from healthier items post‐intervention compared with baseline.

Four studies combined availability of healthier options with information, positioning or pricing. Dorresteijn et al. [75] increased availability and prominence, adding signs to promote healthier choices. Authors reported no change in behaviour with promotional signage, but a sevenfold decrease in the purchase of margarine when the relative positions of two interchangeable items (margarine and butter) were reversed moving butter to central locations and placing margarine in a distant position. Prospective data collection, consideration of confounding factors, and time‐series analysis reduce risk of bias. A second study implemented similar changes but due to combined implementation limited conclusions can be drawn about individual components [77]. A third study changed availability and position of snacks on shelves [76]. Changing the shelf position had no significant effect on purchasing, however, more healthy purchases occurred when the proportion of healthy items was increased from 25% to 75%. Finally, a multi‐component canteen study modified menus to increase availability of low salt, unprocessed items, moved salt to “on request”, offered free extra salad and vegetables with meals, and displayed nutritional information on salt and healthy diets [90]. Twenty‐four hour dietary recall supplemented observer‐recorded canteen choices to estimate nutritional intake, demonstrating no compensatory increase in intake at other points in the day. Compared with control, the intervention group had significantly lower intake of sugar, fat, saturated fat and salt. The cross‐sectional design cannot eliminate the possibility of reverse causation (health‐conscious staff may choose to select the canteen with healthier choices).

In summary, cafeteria results were generally positive for purchasing or dietary intake whereas biomarkers such as weight were non‐significant. The primary nutritional focus of studies was reducing calorie consumption. Labelling studies commonly included low fat and salt content as desirable criteria but only six studies explicitly aimed to increase consumption of wholegrain, fruit and vegetables [36, 39, 42, 43, 48, 61, 77, 88, 90]. Results suggest that changing the availability, pricing and positioning of items are associated with a change in food choice. Evidence for informational changes was less clear with positive effects for traffic‐light labelling whereas logos, cartoon labelling or health messaging were ineffective and at worst, had a negative reaction, increasing unhealthy purchasing. None of the cafeteria‐based studies used sizing, presentation, functionality or defaults.

### 3.3 Hospital Vending and Retail

Eight studies examined hospital vending machines [54, 63, 64, 65, 66, 67, 80, 92]. Pechey et al. [64] varied the relative and absolute number of healthy options. Energy purchased decreased with reduced unhealthy item availability. Sales were maintained despite a restricted number of different items available for purchase. This was consistent with a PHE study [67]. Drinks sales increased while energy per drink purchased decreased by 36% when machines were stocked with 80% low‐calorie beverages. A further four studies on availability support these findings [54, 65, 80, 92]. One UK hospital altered labelling, availability and positioning [63]. Traffic‐light labelling was associated with a small significant reduction in red‐labelled food and drink sales (2.5% and 5%, respectively). Increased healthy item availability resulted in a 36% reduction in red‐labelled purchases compared to control. One study reported a back‐fire effect of a “credible” logo [66]. Healthy products were labelled with low‐credibility (“lighter choices”), high‐credibility (NHS logo) or no label (control). The NHS logo was associated with predominantly more unhealthy sales.

Six studies examined hospital retail outlets [59, 60, 68, 69, 84, 91]. A cluster RCT of 30 hospital shops displayed eye‐level signs ordering single‐serve snacks from lowest to highest calorie from left to right [68]. Purchasing data demonstrated a small but significant reduction from baseline in calories purchased (1.84 kcals, 95% CI: −0.83 to −2.85; *p* < 0.001) at intervention sites while maintaining sales. Two studies implemented availability, information, positioning, pricing and sizing interventions [69, 91]. Neither study implemented a control. Kawabata et al. [91] used a baseline survey and the EAST framework to develop the intervention, the only included study to explicitly use a behaviour change framework to inform the intervention approach. Multiple simultaneous changes and proxy measures of intake could overestimate the impact on dietary change, however, both studies found a consistent direction of effect, supporting the RCT above.

Pricing interventions were associated with purchasing changes without reducing revenue. A 20% increase in price of unhealthy beverages (based on macronutrient and energy content) resulted in a 27.6% reduction in unhealthy beverages (95% CI: −32.2 to −23) and a 26.9% increase in healthy beverages (95% CI: 14.1 to 39.7) [84]. A second study taxing unhealthy items found that while labelling increased the probability of a healthy purchase by 6% (95% CI: 0.28%, 11.80%, *p* = 0.04), a 30% tax with or without labelling increased the probability of a healthy purchase by 10% to 12% (*p* < 0.001) [60].

### 3.4 Inpatients

Five studies examined inpatient food [71, 72, 81, 85, 89]. An RCT redesigned the paediatric inpatient menu labelling recommended frequency (healthier choices were encouraged more often), prominently positioning healthier items and introducing cartoon characters to encourage fruit and vegetable selection [85]. These interventions were associated with an increase in healthy choices, a decrease in unhealthy choices, and positive but non‐significant increased selection of fruits and vegetables. An alternative approach in Denmark created a dining room, removed disease‐related posters, provided tablecloths, napkins and played music resulting in high levels of patient satisfaction [89]. Dietary intake data showed that a significantly higher proportion of patients reached the 75% threshold for nutrient requirements compared to baseline.

An Australian study replaced paper with digital menus including photographs, nutritional information and between‐meal ordering [81]. Digital ordering increased both energy and protein intake. One study introducing a room service system did not improve weight or handgrip strength although short length of hospital stay could be insufficient to see changes in biomarkers [72]. A controlled study using standardised verbal prompts when patients ordered meals by telephone resulted in more frequent selection of high protein items [71].

These studies demonstrate that modifying the food environment can increase energy and protein intake with high levels of inpatient satisfaction. Unlike transient staff encounters, inpatient studies could measure dietary intake and biomarkers and influence 24‐h choices. However, none of the adult inpatient studies considered wider assessment of diet quality against national guidelines or opportunities to normalise healthier long‐term eating habits.

### 3.5 Nursing Homes

Three nursing home studies examined food presentation aiming to increase overall food intake [57, 58, 93]. Neither increasing the availability of meals highly‐rated by residents [57] nor buffet‐style dining and environmental changes [58] altered designated outcomes. Hansen et al. [93] found that offering food on contrastingly coloured dinner plates to 12 nursing home residents modestly increased meal completion but small sample size and risk of confounding limit generalisability. Nursing homes are an under‐researched area.

### 3.6 Multiple Worksites or Food Providers

Five studies [53, 62, 70, 73, 74] included other worksites outside a health setting. Data for healthcare settings were not analysed separately so limited conclusions can be drawn on effectiveness specifically for hospital staff and visitors. Three studies were conducted in the Netherlands, one in the UK, and one in the US. Four studies examined dietary intake and one study looked at purchasing data. All these interventions exclusively focused on staff.

Four hospital studies [55, 56, 82, 83] looked at more than one food outlet at the site. Two before and after studies [82, 83] used pure choice architecture interventions, reducing availability of SSBs in cafeteria, vending, and retail outlets. Both studies noted an increase in proportion of diet or low‐sugar beverages and a decrease in SSBs purchased. Neither study formally assessed compensatory behaviour although survey results suggested that some individuals had changed to bring drinks in from home. Two studies [55, 56] implemented other interventions alongside choice architecture. Epel et al. [55] reduced availability of SSBs from cafeterias, vending machines, food services and retail alongside health education and personalised motivational materials. LaCaille et al. [56] implemented calorie‐based traffic‐light labelling, increased availability of healthy items, reduced the size of serving spoons and reduced visibility of unhealthy foods in the cafeteria and hospital vending machines. This was part of a larger intervention which promoted physical activity and educational messaging on calorie content and traffic‐light labelling. Outcome measures of weight and BMI for this study did not show a significant change from baseline, nor in comparison to the control group. The three studies that reduced SSB availability demonstrated a significant reduction in sales and Epel et al. [55] reported an additional significant reduction in some measures of adiposity at 10‐month follow up [55, 82, 83].

## 4 Discussion

This review found evidence to support using choice architecture interventions to encourage healthier food choices in hospital settings. However, there is limited research on interventions encouraging plant‐based diets or measuring environmental impact and very few studies were conducted in inpatient or care home settings. Creating a choice environment that encourages healthy plant‐based diets has potential to benefit the health of staff, nutritionally‐well patients, and wider society and reduce healthcare costs through reduced prevalence of diet‐related diseases. However, comprehensive evaluation of this approach is needed.

Of the choice architecture typologies identified in this review, changes to availability, pricing and positioning of items appear effective at changing food choice, whereas evidence for informational changes was mixed. Negative or ineffective informational changes were observed with logos or cartoons, this could relate to unanticipated negative association of images, altering desirability e.g., The NHS logo, though credible, doesn't immediately bring appetising food to mind. Few studies examined sizing or presentation, and none implemented changes to functionality or defaults; research is needed to determine effectiveness of these typologies in health and care settings. None of the studies explicitly aimed to encourage a plant‐based dietary pattern and only 12 of the studies included a measure of plant‐source foods such as fruit and vegetable servings. Choice architecture studies to increase plant‐based choices have been conducted in education settings, however, hospital staff and patients may differ in environmental and health literacy and so these results may not be generalisable [94, 95]. Likewise, implementing a plant‐based default option has been shown to be effective in RCTs conducted in education or workplace settings [96, 97].

For hospitals, inadequate evaluation of nutritional outcomes prevented inclusion in this review, however, grey literature suggests that plant‐based hospital menus are of great interest internationally [98]. Vancouver General Hospital has piloted a planetary health menu with new patient‐approved meals that is currently undergoing data analysis [99]. In Germany, a central production unit provides plant‐based meals for retirement homes and hospitals using tailored branding to increase appeal to the recipient [100]. Unfortunately, other than anecdotal reports of public acceptability, the approach has not evaluated nutritional outcomes. Similarly, the initiative reported in New York clearly demonstrates feasibility, acceptability (patient satisfaction), and financial viability of using default meals to increase plant‐based choices but lacks measurement of nutritional and health outcomes – crucial measures for wider adoption given the risk of malnutrition in inpatient populations.

Studies that evaluated a measure of financial viability found in general that sales were maintained or even increased. This is particularly important for retail where profit margins are closely monitored. While none of the studies examined plant‐based diets, the modelled health benefits and resulting healthcare costs of shifting diets have been assessed [101] [98]. Future studies should include economic evaluation of hospital initiatives to shift dietary behaviours.

An expert convention on optimal marketing of plant‐based diets recommends avoiding the terms meat‐free, vegan, vegetarian, or health restrictive language (e.g. low fat) and instead to highlight provenance, flavourful terms (zesty, rich, slow‐roasted), and the food's look and feel [102]. None of the included studies made use of this concept, opting instead to focus on labelling and colour coding foods. Similarly, choice architects can struggle to adequately communicate aims and might be criticised for subverting choices rather than supporting health [103]. Turetski et al. [104] found that defaults were least acceptable although this appears inconsistent with hospital projects where a default approach to plant‐based diets achieved high patient satisfaction ratings [98]. Further research is required in health and care settings to determine whether carbon labelling or environmental messaging might change purchasing behaviours: evidence from eco‐labelling field studies is yet to demonstrate effectiveness [105, 106].

Plant‐based options may apply differently to staff and inpatients. With few inpatient studies, it is unclear whether choice architecture interventions that are effective in staff would be generalisable to patients. BDA recommendations on protein and energy for nutritionally vulnerable patients differ for nutritionally well patients; future studies need to closely monitor nutritional impact for each group. Similarly, in care homes, food choice architecture could be tailored to support nutritional needs of the differing age demographic, cultural preferences, and prevalent cognitive impairment of residents. Well‐designed studies are needed in both settings where levers for behaviour change and nutritional requirements may differ from the general population.

Limitations of this study include that screening was carried out by a single reviewer (this study formed part of an MSc thesis) and, therefore, study inclusion may be less reliable than if independent screening from a second reviewer had been possible. Additionally, no formal assessment was conducted for risk of bias. Previous reviews have assessed study quality and risk of bias with choice architecture interventions and detail common methodological weaknesses that should be considered in future research [27].

Conclusions are limited by variation in study quality due to absence of controls, power calculations, randomisation, or data on potential confounders. Social desirability may bias self‐reported questionnaires, and few studies assessed compensatory behaviour with 24‐h food diaries. However, the consistent direction of effect suggests that pricing interventions in hospital retail can have a large impact on purchasing behaviour. Labelling and positioning prompts had a small but positive effect on healthy purchasing and did not negatively affect commercial viability. The back‐fire effect observed using a health provider logo demonstrates the importance of considering how information is interpreted when making changes. A meta‐analysis of choice architecture interventions found moderate publication bias with overrepresentation of positive studies [107]. Behaviour change frameworks are underutilised. Evidence suggests food choices are particularly sensitive to choice architecture with larger effect sizes than other domains, making this a promising lever for behaviour change to support healthier diets [107]. Frameworks can help classify and tailor interventions to the setting. Theory‐based interventions may be more effective by addressing the underlying causes of behaviours [108]. However, only one study in this review used a framework to design and implement changes [91]. Additionally, long‐term follow up is lacking, particularly for inpatients studies, making it unclear whether changes in dietary behaviours persist after discharge.

## 5 Conclusion

This review demonstrates that choice architecture interventions are effective tools for promoting healthier food choices in health and care settings. However, the current evidence base on interventions that encourage plant‐based diets is insufficient and demands greater expansion. Future interventions must be strategically designed using established behaviour change frameworks and tailored to specific local needs to reveal clear causal pathways. Researchers and practitioners should prioritise the implementation and evaluation of choice architecture strategies in inpatient and care home settings, with explicit focus on nutritional quality, environmental sustainability, and cost‐effectiveness. Interventions promoting plant‐based diets should be assessed not only for immediate dietary changes but also for long‐term health outcomes. Crucially, longitudinal follow‐up is essential to determine whether behaviour change is sustained, quantify broader healthcare, societal, and environmental benefits, and inform scalable, impactful policy and practice.

## Author Contributions

Conceptualisation: Victoria Bion. Methodology: Victoria Bion and Grace Turner. Investigation and analysis: Victoria Bion. Resources: Victoria Bion and Grace Turner. Writing original draft: Victoria Bion. Review and editing: Victoria Bion and Grace Turner. Supervision: Grace Turner. All authors read and approved the final version of the manuscript.

## Conflicts of Interest

VB is affiliated with Real Zero, a nonprofit organisation focused on climate change and health. However, this affiliation has not influenced the study's design, analysis, or conclusions. The authors declare no conflicts of interest.

## Peer Review

The peer review history for this article is available at https://www.webofscience.com/api/gateway/wos/peer-review/10.1111/jhn.70111.

## Data Availability

Data sharing is not applicable to this article as no new data were created or analyzed in this study.

## Appendix 10

Table A1

**Table A1:** Search strategy used to interrogate MEDLINE.

#	Searches	# of hits
20	limit 19 to (english language and humans and yr = “2000 ‐Current”)	1941
19	6 and 12 and 18	2289
18	13 or 14 or 15 or 16 or 17	2,509,696
17	healthcare.mp.	421,665
16	residential facilit*.mp. or exp residential facilities/or residential care.mp. or nursing home*.mp. or exp nursing homes/	75,762
15	home care services.mp. or exp home care services/	52,475
14	exp hospitals/or hospital*.mp.	2,080,454
13	exp patients/	86,752
12	7 or 8 or 9 or 10 or 11	1,643,505
11	catering.mp.	2373
10	(plant‐based or vegetarian* or vegan*).mp.	18,152
9	exp meals/	9305
8	exp food/	1,522,439
7	diet/or exp diet, plant‐based/or exp diet, mediterranean/or exp diet, vegetarian/or exp diet, vegan/or exp dietary patterns/or exp diet, healthy/	206,521
6	1 or 2 or 3 or 4 or 5	708,400
5	choice architecture.mp. or exp decision making/	239,575
4	nudg*.mp.	2577
3	exp Choice Behavior/or choice behavio?r.mp. or choice intervention.mp. or exp Food Preferences/or food preference*.mp.	79,172
2	exp Health Behavior/or Health Behavio?r.mp. or behavio?r intervention.mp.	380,102
1	exp Health Promotion/or health promotion.mp.	113,096

## Appendix 11

Table B1

**Table B1:** Preferred Reporting Items for Systematic reviews and Meta‐Analyses extension for Scoping Reviews (PRISMA‐ScR) Checklist.

SECTION	ITEM	PRISMA‐ScR CHECKLIST ITEM	REPORTED ON PAGE #
**TITLE**			
Title	1	Identify the report as a scoping review.	1
**ABSTRACT**			
Structured summary	2	Provide a structured summary that includes (as applicable): background, objectives, eligibility criteria, sources of evidence, charting methods, results, and conclusions that relate to the review questions and objectives.	3–4
**INTRODUCTION**			
Rationale	3	Describe the rationale for the review in the context of what is already known. Explain why the review questions/objectives lend themselves to a scoping review approach.	5–6
Objectives	4	Provide an explicit statement of the questions and objectives being addressed with reference to their key elements (e.g., population or participants, concepts, and context) or other relevant key elements used to conceptualize the review questions and/or objectives.	7
**METHODS**			
Protocol and registration	5	Indicate whether a review protocol exists; state if and where it can be accessed (e.g., a Web address); and if available, provide registration information, including the registration number.	n/a
Eligibility criteria	6	Specify characteristics of the sources of evidence used as eligibility criteria (e.g., years considered, language, and publication status), and provide a rationale.	7–8
Information sources	7	Describe all information sources in the search (e.g., databases with dates of coverage and contact with authors to identify additional sources), as well as the date the most recent search was executed.	7
Search	8	Present the full electronic search strategy for at least 1 database, including any limits used, such that it could be repeated.	31 (Appendix 10)
Selection of sources of evidence	9	State the process for selecting sources of evidence (i.e., screening and eligibility) included in the scoping review.	7–8
Data charting process	10	Describe the methods of charting data from the included sources of evidence (e.g., calibrated forms or forms that have been tested by the team before their use, and whether data charting was done independently or in duplicate) and any processes for obtaining and confirming data from investigators.	7–8
Data items	11	List and define all variables for which data were sought and any assumptions and simplifications made.	8, 32–50 Appendix 3
Critical appraisal of individual sources of evidence	12	If done, provide a rationale for conducting a critical appraisal of included sources of evidence; describe the methods used and how this information was used in any data synthesis (if appropriate).	n/a
Synthesis of results	13	Describe the methods of handling and summarizing the data that were charted.	8
**RESULTS**			
Selection of sources of evidence	14	Give numbers of sources of evidence screened, assessed for eligibility, and included in the review, with reasons for exclusions at each stage, ideally using a flow diagram.	8–9 (Figure 1)
Characteristics of sources of evidence	15	For each source of evidence, present characteristics for which data were charted and provide the citations.	32–50 Appendix 3
Critical appraisal within sources of evidence	16	If done, present data on critical appraisal of included sources of evidence (see item 12).	n/a
Results of individual sources of evidence	17	For each included source of evidence, present the relevant data that were charted that relate to the review questions and objectives.	8–19
Synthesis of results	18	Summarize and/or present the charting results as they relate to the review questions and objectives.	18–19
**DISCUSSION**			
Summary of evidence	19	Summarize the main results (including an overview of concepts, themes, and types of evidence available), link to the review questions and objectives, and consider the relevance to key groups.	20–21
Limitations	20	Discuss the limitations of the scoping review process.	21–22
Conclusions	21	Provide a general interpretation of the results with respect to the review questions and objectives, as well as potential implications and/or next steps.	22
**FUNDING**			
Funding	22	Describe sources of funding for the included sources of evidence, as well as sources of funding for the scoping review. Describe the role of the funders of the scoping review.	22

*From:* Tricco et al. [109] PRISMA Extension for Scoping Reviews (PRISMAScR): Checklist and Explanation.

## Appendix 3 Study characteristics

Characteristics of included studies grouped by setting.

Table C1

**Table C1:** Studies on hospital cafeterias.

Study	Design	Intervention	Outcome measure	Diet promoted
Whitt [36] 2018 USA	Before and After Experimental Study *Comparator:* No comparator (Baseline or washout period)	All Café items were classified, and traffic‐light labelled as healthy, neutral or unhealthy criteria. For the cartoon labelling, only healthy items (which formerly received green stickers) received a SpongeBob SquarePants quarter‐sized sticker. Flyers describing each labelling system were posted in Café.	Purchasing: Proportion of daily healthy, neutral and unhealthy purchases.	Healthy: fruits or vegetables, whole grain, lean protein or low‐fat dairy; Negative criteria: (i) saturated fat > 5 g per entrée or > 2 g per item and (ii) > 500 calories per entrée, > 200 calories for item or > 100 calories per condiment or beverage.
Ryan [78] 2020 Australia	Before and After Observational study *Comparator:* No comparator (Baseline)	Unhealthy ‘red’ beverages were removed from display and stored behind the counter without this change being communicated to customers, although unhealthy beverages were still available upon request. Beverages were categorised based on the Victorian Government ‘Healthy Choices—customers were not aware of classifications.	Purchasing: weekly sales	Red: sugar‐sweetened soft drinks, fruit juices with added sugar, sports drinks and energy drinks. Amber: zero‐energy and low‐energy ( < 300 kJ per serving) soft drinks, and small ( ≤ 250 mL) ≥ 99% fruit juices. Green: Small ( ≤ 300 mL) reduced‐fat flavoured milks, water and sparkling water.
Thorndike [37, 38, 41, 44, 45] 2021–2023 USA	RCT (individual) *Comparator:* Control group that did not received feedback or incentives	IG received weekly personalised emails (2 per week) informed by cafeteria purchases, baseline questionnaires about diet, physical activity and health goals. 1st email: feedback on previous weeks colour category for purchases, number of calories purchased compared to the recommended daily caloric intake. 2nd email: diet and exercise tips. IG also received monthly letters with peer comparisons (% green purchases compared with all or healthiest employees) and financial incentives Both control and IG received 10% discount on cafeteria purchases.	Both purchasing and biomarkers: Healthy purchasing scores (HPS) were calculated by weighting colour categories; Healthy eating index (HEI‐2015) scores were calculated from two 24‐h dietary recalls.	Low fat and calorie, high vegetable, fruit or lean protein for green‐labelled foods
Thorndike [40] 2016 USA	RCT (individual) *Comparator:* Control group that did not received feedback or incentives	A three‐arm randomized trial comparing (1) social norm feedback letters about the employee's healthy cafeteria purchases; (2) social norm feedback plus small financial incentives to increase healthy food purchases; and (3) no feedback or incentives (control) over 3 months. The feedback‐only arm received four letters over 3 months.	Purchasing: The main outcome was change in proportion of green‐labelled purchases at the end of 3‐month intervention. Post hoc analyses examined linear trends.	Low fat and calorie, high vegetable, fruit or lean protein for green‐labelled foods
Levy [39, 42, 43, 61] 2012–2019 USA	Interrupted time series observational study *Comparator:* No Comparator (baseline)*	A traffic‐light–labelling intervention (in which green indicates healthy, yellow indicates less healthy, and red indicates least healthy) was put in place along with signs explaining the change. Three months after this introduction further changes included rearranging beverage refrigerators, chip racks, and premade sandwiches to have the healthiest choices at eye level and placing baskets of bottled water near food stations.	Purchasing: Of items bought: % red, % green (red labels and green labels as denoting unhealthy and healthy choices, respectively). Of beverages bought: % red, % green.	Low fat and calorie, high vegetable, fruit or lean protein for green‐labelled foods
Block [46] 2010 USA	Non‐randomised controlled trial *Comparator:* Control site (for 3 of the phases)	Price increases were made to SSBs by analysing studies on the price elasticity of demand for regular soft drinks. An educational message was implemented using the energy calculation that 1 pound of weight loss requires a 3500‐calorie deficit—decreasing intake by 1 bottle daily could lead to a 25‐pound weight loss after 1 year if no other change in diet or activity occurred	Purchasing: Number and category of drinks purchased per day and total number of beverage sales.	Reduction in SSBs: Sugar‐sweetened beverages (could be defined as sodas, sports or energy drinks, “fruit” drinks, and sweetened bottled teas and coffees ‐ not from paper) In paper just referred to as “regular soft drinks” versus zero sugar drinks
Lowe [47] 2010 USA	Randomised control trial *Comparator:* Environmental Change only Group	For both groups, 2 environmental changes (EC) were implemented. (1) Energy density of some foods reduced. (2) Introduction of nutritional labels for all foods sold in the cafeterias (previously labels were on less than 10% of foods). Participants in the EC‐Plus condition received two additional components: training in reducing the Energy density of their diet and discounts on low energy density foods purchased.	Both Purchasing & Dietary Intake: Fruit, vegetables, bread dairy, fats and sweets and meats (servings/day) Purchased kcal (kcal): from fat (%); from protein (%); from carbohydrate (%). 24‐h dietary recalls at 4 time points.	Low energy density foods encouraged: The food labels contained a colour coding system which identified each item as very low in energy density (< 0.6 kcal/g, green), low‐energy density (0.6–1.5 kcal/g, yellow), medium energy density (1.6–3.9 kcal/g, orange), or high energy density (4.0–9.0 kcal/g, red).
Warsaw [48] 2020 USA	Before and After Experimental Study *Comparator:* No comparator (baseline)	Introduction of a pricing strategy that reduced the prices of the salad bar and bottled water while increasing the price of cheeseburgers. The hospital cafeteria decreased the salad bar price from $8/lb to $4.99/pound and bottled water from $1 to $0.75. 10 months later, the price of cheeseburgers was increased from $4.25 to $5.50	Purchasing: biweekly point‐of‐sale data for the Four Lakes Café—the largest food retail space.	Promoted salad and water and aimed to decrease cheeseburgers
Sato [49] 2013 USA	Interrupted Time Series Experimental Study *Comparator:* No comparator (baseline)	Labelling and availability of healthy entrees. Healthy pick (HP) entrées were analysed and modified as needed to contain 35% calories from fat or less, less than 10% calories as saturated fat, and less than 1,000 mg of sodium per entrée. Regular menu (RM) entrées were standardized, but no changes were made to the recipes. Two entrée choices were featured each day: one HP entrée and one RM entrée. The meals were labelled with nutritional information on calories, fat, and sodium.	Purchasing: Sales of health versus routine entrees	Reduced salt, fat and calories
Mah [87] 2023 Canada	Interrupted Time Series Experimental Study *Comparator:* No comparator (baseline)	In the Snacking Made Simple intervention, the five healthier items received a −13% to −25% discount, and the five less healthy items received a concurrent price increase of +13% to +26%. The price intervention was consistent at all outlets. Other aspects were specific to each of the four outlets	Purchasing: Weekly purchase differences between healthier and less heathy targeted items	Rationale for what is considered healthy not specified: Healthy items: Fruit, bottled Water, milk. Unhealthy: Loaf Cake Slice, Mini Cinnamon Bun, Rice Krispie Square, Milk Chocolate
Meeusen [77] 2023 Netherlands	Before and After Experimental Study *Comparator:* No comparator (baseline)	Four nudging strategies. (1) Placement: healthy choices at the front, unhealthy choices at the back: criteria based on the Dutch Healthy Food Guidelines. (2) Product: increase ratio of whole‐wheat bread ratio 75% vs non‐whole‐wheat bread 25%. (3) Product: introducing a yoghurt bar in compliance with the Dutch Healthy Food Guidelines. (4) Promotion: signage with information emphasising the health benefits of the products offered, as well as encouraging statements.	Purchasing: Nutritional value, type of product, purchased products via photographed lunch trays	Dutch Healthy Food Guidelines for classifying these products as healthy or unhealthy. Promoted items such as wholewheat bread, low‐fat yoghurt, quark, fresh fruit, unsalted nuts and seeds, high fibre, high vegetable intake
Mazza [50] 2018 USA	Interrupted Time Series Experimental Study *Comparator:* No comparator (baseline/first 3 phases of the study which included pricing and labelling)	A phased intervention over 21 months. Most intervention phases lasted roughly 15 workdays. The first three interventions were implemented cumulatively during the first three phases and remained in place throughout the entire study. Combined, these three interventions constituted the “control conditions.” The remaining phases introduced changes that were removed for washout periods. These included adding emoticons, health messages (about calories consumed, exercise requirements to burn calories, and % of daily calories), social norm feedback (fraction of cafeteria patrons choosing healthy beverages and healthy chips), oppositional pairing (adding a healthy substitute), and colour grouping in positions using the traffic‐light labelling	Purchasing: Sale of beverages and chips	Reduced consumption of regular soda (beverage), calorie reduction, labelling based on criteria for reduced calorie, fat, cholesterol, sodium, saturated fat, trans fat, sugar to be a “healthier” item
Webb [51] 2011 USA	Nonrandomised control trial *Comparator:* Control cafeterias *n* = 2	A 12‐week pilot program of calorie labelling of menu choices in addition to the “Healthy Picks” program already in place in which a logo identified the healthiest choices. Two different menu labelling interventions were designed: (1) calorie information was posted on countertop menu boards at the consumer point of decision and nutrition information including calories was listed on a poster in a central location in the cafeteria, away from the point of decision. For beverages, a highly visible sign was placed on the door of one of the beverage cold cases; (2) calorie and nutrition information was provided only on posters placed away from the point of decision. No calorie information was posted at the 2 control sites.	Purchasing and customer awareness	Calorie content used to classify items as desirable or non‐desirable for the intervention
Patsch [52] 2016 USA	Before and After Experimental Study *Comparator:* No comparator (baseline)	During the 3‐month baseline phase new healthy items were added to cafeteria menus without marketing and pricing components. During the 9‐month Better Bites (BB) intervention phase marketing and direct price incentives/disincentives were introduced. Point‐of‐ purchase marketing included (1) BB logo on all food items meeting nutritional criteria (2) signage highlighting taste, cost, and health benefits. A 35% intervention price differential was used.	Purchasing: Average weekly healthy (Better Bites) and less healthy sales, change in the proportion of healthy and less healthy products sold and financial outcomes.	Nutritional Criteria for better bites items: reduced calorie, reduced total and saturated fat, higher fibre, lower sodium.
Vanderlee [86] 2014 Canada	Cross‐sectional Observational study *Comparator:* Control cafeteria	The intervention site featured energy (calorie), sodium and fat content on digital menu boards, as well as a health logo for ‘healthier’ items. A revised menu improved the nutrient profiles and removed the deep fryer from the cafeteria. The ‘control’ site provided limited nutrition information at the point of sale.	Purchasing: Self‐reported food purchases and nutritional analysis of these foods	Lower energy (calories), sodium, saturated fat and total fat
van Kleef [76] 2012 Netherlands	Before and After Experimental Study *Comparator:* No comparator	Snack options were organized in a four row ‘table’ with four snacks appearing in each row. (1) 25% of products healthy, these located at top shelves. (2) 25% of products healthy, these located at bottom shelves. (3) 75% of products healthy, these located at bottom shelves. (4) 75% of products healthy, these located top at shelves.	Purchasing: Sales (daily)	Selection of 12 relatively healthy and 12 unhealthy snacks based on guidelines from the Netherlands Nutrition Centre (governmental funded organization re‐ sponsible for public nutrition education
Geaney [90] 2011 Ireland	Cross‐sectional Observational study *Comparator:* Control cafeteria	All menus were modified to ensure that the healthiest option was available. Purchasing orders for high‐salt products (gravy mixes, stock cubes) and processed meat (bacon, corned beef) were replaced with low‐salt options (turkey, chicken and fish). Salt was removed in all cooking processes. In the staff canteen, salt was removed from the tables, but small salt sachets were available. Nutrition information on salt reduction and a healthy diet was displayed in the canteen area. No sauces or accompaniments were added to any meals without the customer's consent. Staff were encouraged to consume extra salad and vegetable options with no extra cost. Cooking methods with oil were limited. All desserts were fruit‐based.	Dietary Consumption: 24‐h dietary recall of foods	Restriction of food high in salt, fat and sugar. Promoted higher vegetable intake
Dorresteijn [75] 2013 Netherlands	Interrupted Time‐series Experimental Study *Comparator:* no comparator (baseline)	4 interventions using prompts for: (1) stair climbing promoted by signs placed above elevator buttons. (2) restaurant cream soup salt content reduced by 30%, signs at counter promoting reduced salt content. (3) lean croissants introduced with 30% less fat and 20 few kcal. (4) Diet margarine in easy to reach positions and butter cups placed further away in a fridge.	Purchasing only: Number and ratio of purchased normal‐salt soup, reduced‐salt soup, croissants, lean croissants purchased, diet margarine and butter.	Reduced salt, reduced fat and calorie foods
Lassen [88] 2014 Denmark	Nonrandomised controlled trial *Comparator:* No intervention control canteen	Introduction of healthy Nordic Keyhole symbol on freshly prepared food. The goal of the intervention canteen was that at least half of the meals should be healthy labelled.	Dietary consumption: (Not 24 h, for the meal encounter only) Energy per meal (kJ), energy density (kJ/100 g), fat content (E%), fruit and vegetables (g/100 g), salt (g/100 g), refined sugars (g/100 g), wholegrain (g/100 g).	Criteria for the maximum amounts of fat, salt and sugars, together with the minimum amount of dietary fibre and wholegrain in 25 different food groups.
MacDonald [88] 2016 Australia	Interrupted time series Observational design *Comparator:* No comparator (baseline)	Nutrition resources developed to guide healthy eating policy implementation by caterers at each site. This included order forms with a ‘traffic‐light’ system distinguishing food categories based upon nutrient composition that designated that at least 50 per cent of a catering order should comprise food and drinks from the ‘best choice’ category, and no more than 20 per cent from the ‘limit’ category. Distribution of traffic‐light guide posters and information sheets to catering staff.	Purchasing: Proportion of purchased foods in category ‘foods to limit’ (%).	Green‐labelled foods: low saturated fat, sugar, salt, calorie, and high in fibre. Red foods: high in energy, saturated fat, sugar, salt and low in fibre. Increased fresh fruit, low fat milk, low sugar.

*Note:* Study characteristics are presented from hospital cafeteria studies including details on design, intervention, outcome measures used, and the type of diet the study aimed to promote. IG = intervention group, SSB = sugar sweetened beverage. *2012 study used the 2 on‐site cafeterias as comparator.

Table C2

**Table C2:** Studies on hospital vending.

Study	Study design	Intervention	Outcome	Diet promoted
Gorton [92] 2010 New Zealand	Before and After Experimental Study *Comparator:* No comparator (baseline)	Threshold criteria for vended foods in 14 hospital vending machines were developed from guidelines (developed by eight nutrition professionals) Two levels of classification were developed: ‘better’ and ‘other’ choices. Vending machines were stocked with 50% better choices and 50% other choices.	Purchasing: nutrient content, sales and staff surveys. Primary outcome measure: energy density (kJ/100 g) and energy per packet (kJ). Secondary Outcomes: sales of total fat, saturated fat, sugars and sodium per 100 g of food sold; change sales and staff satisfaction	Criteria: focused on energy, saturated fat, sodium, and portion sizes of confectionery. To be a ‘better choice’ item, foods were required to contain ≤ 800 kJ per packet, ≤ 1.5 g saturated fat per 100 g, ≤ 450 mg sodium per 100 g, and not be confectionery. ‘Other choice’ items were only required to meet the energy criteria (≤ 800 kJ/packet).
Grivois‐Shah [54] 2018 USA	Before and After Experimental Study *Comparator:* No comparator (baseline)	The intervention consisted of converting the composition of all vending machines across 23 locations from approximately 20% “right choice” (RC) to 80% RC items. Nutrition standards developed by the study design team identified the RC program provided by the vendor as an appropriate intervention.	Purchasing: monthly aggregates of sales, total units vended, calories, fat, sodium, and sugar vended by site	The “right choice” included snacks that have < 10% of calories from saturated fat, zero trans‐fat, < 60 mg cholesterol, < 35% sugar by weight, < 270 mg sodium, and < 200 calories per serving. Right choice beverage selections contain < 25% calories from sugar, < 230 mg of sodium, and < 160 calories per serving.
Campbell [63] 2021 UK	Before and After Experimental Study *Comparator:* No comparator (baseline)	Nutritional traffic‐light labelling; Substitution of products for the industries healthier range; Placement of healthier products into hotspots. Moving water closer to the eye level was the only option available in the experiment, due to service contract restrictions.	Purchasing: Outcomes (given in %) Red labelled drink/food sold	Reduced energy, saturated fat, sugar and sodium—based on The Rayner (2005) model was developed and applied by the UK Government was used to label products
Pechey [64] 2019 England	RCT Multiple treatment reversal design *Comparator:* alternative conditions	Vending machines randomised to one of two sequences for the seven study periods: ABCADEA or ADEABCA: healthier or less healthier options and the proportions of each were varied over different study periods	Purchasing: total energy purchased (kcal) from drinks or snacks per week	Low calorie or fruit/nuts/seeds; drinks with low sugar or 100% fruit juice. Snacks: > 150 kcal (per package) considered less healthy. Exceptions: fruit, nuts and seeds without added sugar or salt (healthier). Drinks: Any beverage containing ≥ 2.5 g sugar per 100 mL (less healthy) The exception to this rule was 100% fruit juice
Boelsen‐Robinson [80] 2017 Australia	Interrupted Time Series Observational Study *Comparator:* No Comparator (baseline)	Reduced availability of unhealthy items increased availability of health items: The health service adopted the Healthy Choices: food and drink guidelines for Victorian public hospitals These guidelines classify food and beverages into ‘red’, items that should be limited, ‘amber’, items to be chosen carefully, and ‘green’, the best choices. There was basic communication at point‐of‐sale as to the meaning of the classification, and items were labelled with their classification. The health service conducted audits every 3–6 months to ensure compliance. Not stated what baseline proportions were or what they were during data collection.	Purchasing: Sales data + stakeholder interviews	Classification is based on nutrients such as saturated fat, sugar, sodium, fibre, and energy content. The proportion of ‘red’ items available in each vending machine across the health service should be no more than 20% of displayed products, ‘green’ at least 50% of displayed products, and ‘amber’ the remaining.
Griffiths [66] 2024 Wales	Controlled experiment ‐ randomisation method not stated: *Comparator:* no labelling vending machine	Healthy products either given a low source credibility label, “lighter choices” or a high source credibility label e.g. NHS logo	Purchasing: sales volume	Healthy products (e.g., baked crisps, cereal bars) and unhealthy products (e.g., standard crisps, chocolate bars). Healthy snacks satisfied the Welsh Hospital Healthy Vending directive constraints whereas unhealthy products did not.
Griffiths [65] 2020 Wales, UK	Non‐randomised controlled trial; *Comparator*: Between healthy and unhealthy conditions	Two vending machines were manipulated in a 6‐month trial, with a healthy and regular range of products alternated between the two machines every fortnight.	Purchasing: calorific content of project sold, sales volume, and cost/profit, sales of nearby shops	Healthy snacks satisfied the Welsh Hospital Healthy Vending directive constraints whereas unhealthy items did not. Generally, low calorie considered better.
Public Health England [67] 2018 England	Before and After Experimental Study *Comparator*: No comparator (baseline)	The first phase increased the availability of healthier food and drinks by applying nutritional standards (including some of the Government Buying Standards for Food best practice criteria). The second phase altered the placement of products, moving healthier products to more prominent, salient positions within the machines. The two interventions were implemented, tested over 2 distinct phases and compared to baseline data.	Purchasing: number of items sold, mean energy (kJ/kcal) and mean sugar content (g) per product purchased	Criteria: SSBs to be < 330 mL pack size and < 20% of beverages may be sugar sweetened. > 80% of beverages (procured by volume) may be low calorie/no added sugar (including fruit juice and water). Savoury snack sizes of < 30 g. Sweet snacks in the smallest single portion size available within the market and < 250 kcal.

*Note:* Study characteristics are presented from hospital vending machine studies including details on design, intervention, outcome measures used, and the type of diet the study aimed to promote.

Table C3

**Table C3:** Studies on hospital retail.

Study	Study design	Intervention	Outcome	Diet promoted
Racette [59] 2009 USA	Cluster randomised controlled trial: *Comparator:* Health assessments only	The intervention comprised nutrition components, physical activity components, and incentives designed to promote healthy dietary and physical activity behaviours. Specific intervention components included weekly healthy snack cart, monthly lunchtime seminars, monthly newsletters, walking maps, participation cards and participation rewards.	Dietary consumption: Fruit and vegetable intake (servings/day).	Weekly health snack cart items not defined
Kawabata [91] 2023 Japan	Before and After Experimental Study *Comparator:* No comparator (baseline)	The intervention was based on the Hospital Nutrition Environmental Scan (HNES) and incorporated one of the nudge frameworks, EAST, advocated by the Behavioural Insight Team (BIT). Intervention: (1) offering a “healthy set” with a discount at grab‐and‐go; (2) increasing and improving the placement of healthy options (e.g., salads, yogurt, sugar‐free drinks, low‐salt bowl noodles); (3) providing nutrition information in the CVS and monthly newsletters.	Biomarker: Change in salt measured using urine check‐ups, staff food intake eating attitude from surveys	Not overtly stated but the implication from baseline diets was that the study aimed to increase vegetables, fruit, and reduce salt. Healthy options (e.g., salads, yogurt, sugar‐free drinks, low‐salt bowl noodles)
Allan [68] 2020 Scotland, UK	Cluster Randomised controlled trial *Comparator: Control shops using block randomisation controlling for shop size*	Point of purchase prompts (PPPs) displaying products in order from highest to lowest energy content on a sign to be displayed at eye level on shop shelves. The PPP was a sign displaying all of the available single‐serve snacks in order from lowest calorie on the left to highest calorie on the right.	Purchasing: Average energy, fat and sugar content of purchases per day, average cost of each purchase and total number of purchases per day.	Low calorie
Elbel [60] 2013 USA	Non‐randomised Controlled trial *Comparator:* Baseline condition implemented twice	5 phase intervention: (1) a baseline condition with no special labelling or taxation; (2) highlighting “less healthy” in red capital letters on the price tag, with a red box drawn around it; (3) a 30% non‐itemised tax on less‐healthy items (i.e., the price listed was simply 30% higher than in the baseline condition); (4) a 30% non‐itemized tax on less‐ healthy items plus highlighting (as in Condition 2); and (5) a 30% itemised, explicit tax on less‐healthy items (the reason why was stated “30% less healthy tax = $0.33”) plus highlighting with the “less healthy” signage	Purchasing: Number and nutritional quality of purchases.	Researchers defined items that met at least two of three standards in their entirety as healthier, and other items as less healthy. Alabama: (≤ 6.5 g total fat, < 30 g carbohydrates; < 360 mg sodium; drink size ≤ 12 oz, or ≤ 16 oz for water and milk; ≥ 5% daily value of vitamin A, vitamin C, iron, calcium, or fiber); California:(≤ 35% of calories from fat, except nuts and seeds; ≤ 10% calories from saturated fat; ≤ 35% total weight from sugar, except fruits & vegetables; and beverages limited to milk, water, and ≥ 50% fruit juice with no added sweeteners); and prior research studies:( ≤ 150 calories for food, ≤ 50 calories for beverages, ≤ 30% calories from fat, and ≤ 35% total weight from sugar).
Blake [84] 2018 Australia	Interrupted Time Series Experimental Study *Comparator:* No comparator (baseline)	Beverages were classified using a state government framework. Prices of “red” beverages (e.g., non‐diet soft drinks, energy drinks) increased by 20%. Prices of “amber” (e.g., diet soft drinks, small pure fruit juices) and “green” beverages (e.g., water) were unchanged.	Purchasing: Changes in beverage volume, item sales, and revenue compared with predicted sales.	Reduced non‐diet soft drinks, energy drinks)
Simpson [69] 2018 England	Before and After Experimental Study *Comparator:* No comparator (baseline)	Components of the intervention included reducing ‘friction costs’ to make healthier choices more likely by introducing healthier products, limiting the portion size of unhealthy options and reducing promotion of unhealthy options; incentivising healthier choices by substituting healthier for unhealthy choices in the meal deal offers; and improving the salience or prominence of healthier option.	Purchasing: sales data on food and drink at 3 time periods	Reduced consumption of “unhealthy products”—high in saturated fat > 20 g or > 5 g added sugar, > 1/.5 g salt per 100 g.

*Note:* Study characteristics are presented from hospital retail outlet studies including details on design, intervention, outcome measures used, and the type of diet the study aimed to promote.

Table C4

**Table C4:** Studies on hospital inpatients.

Study	Study design	Intervention	Outcome	Diet promoted
Barrington [81] 2018 Australia	Before and After prospective observational point prevalence study *Comparator:* no comparator (baseline)	Mixed intervention ‐ introduced electronic ordering system but also altered menu design: the BMOS (bedside meal ordering system) was available 24 h/day for patients to navigate (directly) through and select their main meals and mid‐meals at any time of the day and place menu selections up to 1 h before a meal. Photographs of the menu items were available on the BMOS. The menu, foods, recipes, main meal choices, short‐order meal alternatives and nutrient composition of foods did not change between the two study cohorts.	Dietary consumption: dietary intake and plate waste using visual scale	Overall intake or plate wastage reduction rather than specific nutritional aim
Holst [89] 2017 Denmark	Before and After Experimental Study *Comparator:* No comparator (baseline)	Environmental changes: Dining room walls were painted, and all information material relating to diseases, such as information about smoking, were removed from the dining room. Tablecloths, small vases, coloured tray mats and napkins were introduced for all main meals. Soothing background music was played during lunch and dinner. A “Welcome‐tray” was provided with a special serving and written materials about food and nutrition.	Dietary consumption: food intake, energy intake, protein intake	Increasing nutritional intake
Doorduijn [72] 2016 Netherlands	Before and after prospective observational study: *Comparator:* Traditional meal service 3 meals a day	Room service: Between 7 AM and 7 PM, patients could order foods and drinks from a menu card by telephone call to trained operators. The operator has access to information including type of diet and previous orders and enters the order in the Menu Management System (MMS). Orders were delivered within 45 min. Patients who required an energy‐ and protein enriched diet could still receive the extra in‐between‐meal snacks high in protein chosen from a special menu card.	Biomarkers AND Purchasing: MUST scores, body weight, qualitative data, food ordering data	Increase food intake
Basak [85] 2019 Canada	RCT ‐ cross over design: *Comparator:* Control menu (randomised by ward – method not described)	Layout of menu with child friendly labelling: Menu items were then organized into green, yellow, and red boxes with labels reading “a great healthy choice,” “choose sometimes,” and “choose once in a while,” respectively. A section titled “Eat Like a Superhero” was created to prime children. A suggested sample breakfast, and lunch and dinner meals with photographs of portion sizes in a plate format. Original cartoon female grapes and male broccoli superhero characters were created to encourage fruit and vegetable selection. None of the menu items changed between the control and intervention menu.	Both Purchasing and dietary consumption: Choice of healthy or unhealthy by traffic‐light system, SSB consumption, intake of fruit and vegetables	The coding of the red‐, yellow‐, and green‐light foods was derived using a combination of tools and guidelines, 3 dietitians classified the menu items based on the level of processing and nutrient content, with particular emphasis on fibre, added sugar, saturated fat, and sodium.
van der Zanden [71] 2015 Netherlands	Non‐randomised control trial: *Comparator:* control day (excluding previous IG patients)	The intervention consisted of a verbal prompt: “Would you like some [target product] with that?”, which was presented to patients by trained telephone operators, after patients finished ordering their lunch; on the consecutive day patients received verbal praise followed by the verbal prompt	Purchasing: Ordering metrics, protein and caloric content	Higher protein

*Note:* Study characteristics are presented from hospital inpatient studies including details on design, intervention, outcome measures used, and the type of diet the study aimed to promote.

Table C5

**Table C5:** Studies on nursing homes.

Study	Study design	Intervention	Outcome	Diet promoted
Crogan [57] 2013 USA	RCT Two group repeated measures design: *Comparator:* control NH	The Eat Right food delivery system: A resident‐centred, multi‐level, two‐component delivery system. Pictorial menus were given to residents to rate the dishes. Nursing home menus were updated for the intervention site if dishes received a median score of < 3.5 Residents made food choices by marking menu (prior day) A steam table was introduced for food service (both intervention and control sites).	Biomarkers: satisfaction, weight	Increasing Food intake
Hansen [93] 2018 Norway	Nonrandomised control trial & semi‐structured interviews with staff. *Comparator:* Day 1 with white porcelain ‐ usual care	This study took place in two units in one nursing home including 12 residents; all diagnosed with dementia but able to eat on their own and without help from care staff. The residents’ behaviour, actions and interactions related to dinner plates with different colour combinations were observed during dinnertime. Types of plates used: Plate A: white well, yellow rim and red ring around the edge; Plate B: yellow well, red lip and red ring around the edge; Plate C: white well, green lip and blue rim on the edge and Plate D: white porcelain on 4 consecutive days	Dietary consumption: Each (uniquely numbered) plate was photographed before it was served to each resident. When the resident finished eating the plate was re‐photographed.	Increasing Food intake
Remsburg [58] 2001 USA	RCT pilot study: *Comparator:* Tray style meal – unclear if environment also altered	Baseline to 3‐month comparisons of the following changes: food served from steam table; selection from a variety of foods; residents own choice; second helpings; maintenance of ideal food temperature and enhanced environment (tablecloths, china, personal decorations, music, adaptive utensils and plates, positioning for social engagement.	Biomarkers: Weight change compared to control, blood markers	Increasing Food intake

*Note:* Study characteristics are presented from nursing home studies including details on design, intervention, outcome measures used, and the type of diet the study aimed to promote.

Table C6

**Table C6:** Studies in other settings.

Study	Study design	Intervention	Outcome measure	Diet promoted
Immink [70] 2021 Netherlands Setting: Office Meetings	Non‐randomised control trial: *Comparator:* Control condition	Study 1: Presentation of the vegetables was manipulated by either offering them in separate bowls by type (tomatoes or cucumbers) or scrambled together in a bowl. Study 2: Portion size of vegetables was varied either presenting individual portions or a sharing bowl. Placement of individual portions near chair whereas sharing bowls in the middle. Slogans on cups promoting the benefits of consumption. Conditions randomised over the meetings using a random number generator function in excel. Study 3: One box of vegetables was provided for each 14 persons. In the conditions with cookies, one cookie for each participant was served. The products were placed in the middle of the meeting tables.	Dietary consumption: Weight of vegetables or food consumed per meeting attendee	Increased fruit and vegetable intake
Epel [55] 2020 USA Setting: Mixed Cafeterias, vending machines, food services, retail	Before and After Observational Study and Randomised control trial *Comparator*: no control (baseline)	SSB sales ban alongside motivational intervention with education. Participants were randomised to the brief motivational intervention immediately met with the health educator for a brief motivational interview. Health educators made brief (approximately 5 min) “booster” telephone calls to revisit goals at 1 week after the baseline visit, 1 month after implementation of the SSB sales ban, and 6 months after implementation of the SSB sales ban.	Both Dietary consumption and biomarkers: Changes in SSB intake, abdominal adiposity, insulin resistance assessment	Reduction in SSBs: Sugar‐sweetened beverages (defined as sodas, sports or energy drinks, “fruit” drinks, and sweetened bottled teas and coffees)
LaCaille [56] 2016 USA Setting: Mixed Hospital cafeteria and vending: main campus versus six primary care clinics	Non‐randomised controlled trial *Comparator:* no intervention control employees (93 employees in 5 primary care clinics)	The intervention involved pedometer distribution, calorie labelling, persuasive messaging, and integration of influential employees to reinforce healthy social norms. Every food item in the hospital cafeteria and vending machines was labelled with calories, number of steps required to burn those calories (e.g., a slice of pizza = 690 calories and 13,800 steps), and a “traffic‐light” colour rating. Other changes included reducing the size of serving spoons (start of study), offering half portions at half price (at month 5), increasing the number of “green light” foods (month 7), moving the dessert case to a less visible area (month 8), establishing a highly visible “grab‐n‐go” cooler offering healthy foods (month 8), and decreasing portion sizes of some foods (month 8).	Dietary consumption & Biomarkers: weight, BMI, waist circumference, physical activity, and dietary behaviour after 6 months and 1 year were primary outcomes.	Calorie reduction: Colour codes were based on energy density, which is the number of kcal per gram of food (green ≤ 1.0 kcal/g; yellow = 1.0 to 2.25 kcal/g; red > 2.25 kcal/g. (green = go, eat in large portions, yellow = caution, eat in moderate portions, red = stop, eat in small portions)
Tinney [82] 2022 Australia Mixed: Hospital Retail outlets and vending machines	Before and after observational study. *Comparator:* No comparator (Baseline)	Removing sugar sweetened beverages (SSBs) from sale in a regional health service.	Purchasing: Drink purchasing patterns were measured by product ordering data. Consumer opinion regarding the intervention, self‐reported purchase and consumption were also explored.	Reduction in SSBs: Sugar‐sweetened beverages.
Walker [83] 2020 Australia Setting: Mixed Children's hospital retail food outlet and vending machines	Before and after prospective observational study *Comparator:* No control (baseline)	Decreased availability of less healthy options: Health services were encouraged to apply the strategy to their relevant facility by improving the range, availability and promotion of healthy bottled and canned beverage options while limiting the availability of less desirable options. Criteria for compliance was < 20% red labelled beverages, < 30% amber, > 50% green beverages.	Purchasing: % and number of items sold	Beverages were grouped into three categories depending on their nutritional profile, in particular, their free sugar content. ‘Green’ beverages: encouraged ‘always’, no free sugars, ‘amber’ beverages: ‘sometimes’ choice (either have no free sugars but are artificially sweetened beverages or contain free sugars but have other beneficial nutrients) and ‘red’ beverages: free sugars, minimal nutritional value, are specified as a ‘limited’ choice.
Kwak [74] 2009 Netherlands Setting: Mixed worksites ‐ 2 hospitals and 12 other worksite cafeterias	Before and after experimental study (non‐randomised): *Comparator:* Control: No intervention.	There was an individual and an environmental component to the intervention. Food related component: food diaries (individual), environment changes in the assortment of food products, workshops, an information wall on the balance between food intake and physical activity, posters or prompts for stair use, and ways to form lunch‐walking and cycling groups. Not specified which component each site took up (free to choose)	Dietary consumption: Of high or low energy or fibre foods (all in servings/day).	Low calorie and low energy density (low saturated fat high unsaturated fat, high fibre)
Vermeer [73] 2011 Netherlands Setting: Mixed worksites ‐ 15 hospitals and 10 other worksite cafeterias	Cluster RCT: Longitudinal randomized controlled trial *Comparator:* No intervention (control sites)	Intervention 1: A smaller portion was offered in addition to the existing portion and proportional pricing was employed. Intervention 2: A smaller portion was added to the assortment and value size pricing (i.e., a lower price per unit for large portions than for small portions) was employed (the price was 80% of the existing size).	Purchasing: Number of large meals sold, number of small meals sold, number of fried snacks sold.	Reduced portion size/calorie intake
Holdsworth [62] 2004 UK Setting: Mixed ‐ two healthcare and 4 other worksite cafeterias	Cross‐sectional: *Comparator:* No intervention (two comparison workplaces)	The Heartbeat Award was awarded to catering establishments fulfilling the following criteria: at least one‐third of the dishes on the menu were ‘healthy choices’; at least one‐third of the eating area was non‐ smoking; at least 30% of food handling staff had received training on hygiene; and the premises complied with food hygiene regulations.	Dietary consumption: Daily intake of food items.	The nutrition goals of the scheme are to reduce total fat, sugar and salt, and increase the availability of fibre‐rich, starchy foods
Beresford [53] 2001 USA Setting: Mixed 6 healthcare facilities 22 other worksite canteens	Cluster randomised controlled trial: *Comparator:* No intervention	The intervention was tailored to worksites. Elements included a kick‐off event, the 5‐a‐Day message being posted on boards in each worksite, more fruit and vegetables becoming part of the menus and the provision of a self‐help manual for every employee. Messages included “Are you short‐changing yourself,” “Do something groundbreaking,” and “5 ways to 5 a Day.” Channels to deliver a message included posters, brochures, table tents, paycheck inserts, flyers, newsletters, food demonstrations, message cards, tip sheets, and a self‐help manual.	Dietary consumption: Fruit and vegetable intake (servings/day).	More fruit and vegetables

*Note:* Study characteristics are presented from other settings (hospital offices, studies of hospitals and other worksites, studies of multiple types of hospital food provision e.g., vending and cafeterias). Details are provided on design, intervention, outcome measures used, and the type of diet the study aimed to promote.
